# Prepubertal Exposure to Tris(2-chloroethyl) Phosphate Disrupts Blood-Testis Barrier Integrity via Ferritinophagy-Mediated Ferroptosis

**DOI:** 10.3390/toxics13040285

**Published:** 2025-04-08

**Authors:** Yonggang Zhao, Mo Peng, Honglei Liu, Xiaoyu Zhang, Dan Fu

**Affiliations:** 1 Environment Monitoring Center of Jiangsu Province, Nanjing 210019, China; 2 Nanjing Shenghong Environmental Technology Co., Ltd., Nanjing 210017, China

**Keywords:** TCEP, BTB, ferritinophagy, ferroptosis

## Abstract

Tris(2-chloroethyl) phosphate (TCEP) is a representative chlorinated organophosphate flame retardant (OPFR) that demonstrates greater persistence than other non-halogenated alkyl or aryl OPFRs. Although TCEP has been shown to accumulate significantly in the environment and contribute to testicular toxicity and spermatogenic dysfunction, the precise underlying factors and mechanisms of action remain unclear. Herein, male ICR mice were gavaged with corn oil, 50 mg/kg body weight (bw) TCEP, or 100 mg/kg bw TCEP from postnatal day (PND) 22 to PND 35. TCEP exposure resulted in the disruption of blood-testis barrier (BTB) integrity and in abnormal testicular development. Considering that Sertoli cells constitute the primary target of toxicants and that TCEP induces oxidative stress in the testis and other organs, we focused on ferroptosis in Sertoli cells. Our findings revealed a significant increase in ferroptosis in the testes and Sertoli cells following TCEP exposure, and we observed functional restoration of Sertoli cell junctions upon treatment with the ferroptosis inhibitor ferrostatin-1. Furthermore, ferritin heavy chain 1 (FTH1) was markedly reduced in TCEP-exposed testes and Sertoli cells. Since nuclear receptor coactivator 4 (NCOA4)-mediated ferritinophagy is essential for the degradation of FTH1, we assessed ferritinophagic activity and found significant upregulation of NCOA4, ATG5, ATG7, and LC3B II/I in TCEP-exposed testes and Sertoli cells. These results strongly suggest that TCEP triggers Sertoli cell ferroptosis by activating ferritinophagy that leads to reduced expression of BTB-associated proteins, ultimately causing BTB disruption and testicular developmental toxicity.

## 1. Introduction

Organophosphate flame retardants (OPFRs) are a class of flame retardants based on organophosphorus compounds, and they are used to enhance the fire resistance of materials and reduce the risk of fire [1]. These compounds are often employed as substitutes for polybrominated diphenyl ethers (PBDEs), which have been strictly regulated due to their toxicity and environmental impact [2]. However, the adverse effects of OPFRs—which have been detected in organisms and environmental matrices (including air, water, soil, and indoor dust)—have drawn increasing attention due to their aggressive production and usage [3,4,5,6].

Epidemiological and animal studies have demonstrated that OPFR exposure is associated with various pathological conditions, including respiratory diseases [7,8], hepatotoxicity [9,10], and impairment of renal function [11,12]. With the rising prevalence of male infertility, exposure to environmental toxicants constitutes one of the principal factors that lead to a decline in sperm quantity and quality [13]. Consequently, reproductive damage caused by OPFR exposure in males has elicited greater attention in recent years. Among the various OPFRs, tris(2-chloroethyl) phosphate (TCEP) manifested adverse effects on male reproductive toxicity [14]; however, the specific factors involved and underlying mechanism(s) of action are unknown.

According to recent evidence, exposure to TCEP induces male reproductive toxicity by triggering oxidative stress and disrupting steroidogenesis [14,15,16]; and these adverse effects have been primarily attributed to TCEP’s impact on Leydig cells. However, whether TCEP induces testicular toxicity by damaging Sertoli cells remains largely unknown. Sertoli cells comprise the only somatic cells within the seminiferous tubules of the testes, and they form the blood-testis barrier (BTB) through tight junctions (TJs), basal ectoplasmic specialization (ES), gap junctions (GJs), and desmosomes. This barrier protects germ cells by preventing toxic and harmful substances from entering the seminiferous tubules, while also blocking the leakage of germ cell-derived antigens that might trigger immune responses [17]. This activity maintains the stability of the spermatogenic microenvironment and serves as a prerequisite for the meiotic division of spermatocytes [18]. Furthermore, Sertoli cells—and particularly the junctional structures between them—are primary targets of environmental toxicants [19]. Thus, we hypothesized that TCEP precipitated testicular developmental damage by disrupting the integrity of the BTB.

TCEP, employed as an alternative to PBDEs, may elicit toxic effects similar to those of PBDEs, and PBDE-47 has been shown to induce spermatogenic dysfunction through ferroptosis in Sertoli cells [20]. Ferroptosis is a type of regulated cell death marked by the buildup of iron-dependent lipid peroxides reaching the lethal levels. However, unlike apoptosis, necrosis, or autophagy, ferroptosis is driven by oxidative stress, specifically through the buildup of reactive oxygen species (ROS) that harm cellular membranes. This process is closely linked to iron metabolism, as excess ferrous iron catalyzes the formation of ROS, leading to lipid peroxidation [20]. Ferroptosis has been implicated in various diseases, such as neurodegenerative disorders, cancer, and organ injuries [21]. However, whether TCEP disrupts BTB integrity by inducing ferroptosis in Sertoli cells remains unelucidated.

Autophagy plays a crucial role in regulating ferroptosis, with NCOA4-mediated ferritinophagy essential to its induction [22]. Iron is sequestered in ferritin complexes, which consist of ferritin-heavy (FTH1) and light (FTL) chains. NCOA4 binds to FTH1, facilitating its transport to the autophagosome. Upon fusion of autophagosomes with lysosomes, ferritin is degraded, resulting in the release of iron. Based on this information, we hypothesized that TCEP exposure disrupted BTB integrity via ferritinophagy-mediated ferroptosis in Sertoli cells.

We herein demonstrated that the enhanced degradation of FTH1 via ferritinophagy is a key factor that drives TCEP-induced ferroptosis in Sertoli cells, thereby disrupting BTB integrity; and that this leads to testicular developmental toxicity.

## 2. Materials and Methods

### 2.1. Animals and Treatments

ICR male mice at postnatal day 21 (PND21, corresponding to the prepubertal stage) were sourced from Guangzhou Ruige Biotechnology Co., Ltd. (Certificate No. SCXK 2023–0059, Grade SPF, Guangzhou, China). The Ethics Committee of Guangzhou Shuiyuntian Biological Technology Co., Ltd. (No. SYT2024102, Guangzhou, China) approved the animal procedures used in this study. They were housed at Guangzhou Shuiyuntian Biotechnology Co., Ltd. under standardized conditions, which included a 12 h light/dark cycle, a temperature maintained at 25 ± 2 °C, and relative humidity of 50 ± 5%. The mice had unrestricted access to standard food and distilled water. A total of 36 mice were randomly divided into three groups: a control group receiving corn oil, a T50 group given 50 mg/kg body weight (bw) of TCEP, and a T100 group administered 100 mg/kg bw of TCEP. From PND22 to PND35, all mice underwent daily gavage with either corn oil (Aladdin, Shanghai, China) or TCEP (NJDULY, Nanjing, China). On the second day after the final gavage, the mice were anesthetized with CO_2_ and euthanized by cervical dislocation. The testes were subsequently collected, with the left one promptly frozen in liquid nitrogen for biochemical analysis, while the right one was preserved in 4% paraformaldehyde (PFA) for histopathological examination.

### 2.2. Testis Coefficient

To evaluate the potential testicular toxicity resulting from TCEP exposure, the testis coefficient (TC) was measured. The testis coefficient is defined as the ratio of testis weight to body weight, expressed as a percentage, calculated using the formula: TC = (testis weight (g)/body weight (g)) × 100%.

### 2.3. Hematoxylin-Eosin (H&E) Staining

Testicular tissue sections, embedded in paraffin and sliced to a thickness of 4 µm, were deparaffinized and rehydrated. They were then stained with hematoxylin for 2–3 min, followed by a brief 5 s eosin staining. The sections were subsequently dehydrated using alcohol in varying concentrations, cleared with xylene, and mounted. Finally, they were observed and photographed using a Nikon microscope. Six testes from each group were randomly chosen for analysis. The percentage of abnormal seminiferous tubules with less than three layers of spermatogenic cells and/or sloughed germ cells was determined by analyzing 100 tubules per testis.

### 2.4. TM4 Cell Culture

The TM4 (Sertoli) cell line was obtained from Wuhan Pricella Life Technology Co., Ltd. (Wuhan, China) and cultured in DMEM/F12 medium, enriched with 5% horse serum (Solarbio, Beijing, China) and 2.5% fetal bovine serum (Procell, Wuhan, China). The cells were kept in a humidified environment with 5% CO_2_ at 37 °C. The treatments were administered for 24 h as follows: controls received DMSO; 500 μM TCEP, or a combination of 500 μM TCEP and 0.5 μM ferrostatin-1 (Fer-1), which acts as a ferroptosis inhibitor.

### 2.5. CCK-8 Analysis

TM4 cells were plated in 96-well plates at a density of 5000 cells per well and exposed to varying concentrations of TCEP for 24 h, each concentration was set up with 6 wells, and the experiment was repeated 3 times. Following the removal of the culture medium, 100 µL of a 10% CCK-8 solution (diluted in serum-free DMEM/F12 medium) was added to each well. The optical density (OD) at 450 nm was measured with a spectrophotometer after a 2 h incubation.

### 2.6. Transmission Electron Microscopy (TEM)

Testes were fixed overnight in 3% glutaraldehyde and subsequently post-fixed in 1% osmium tetroxide. The samples underwent dehydration through a graded ethanol series and were embedded in epoxy resin. Ultrathin sections were prepared and stained with uranyl acetate and lead citrate, followed by observation under a JEM-1400PLUS transmission electron microscope (JEOL, Tokyo, Japan).

### 2.7. Analysis of Ferrous Iron

The ferrous iron content in testicular tissue or Sertoli cells was measured using an iron assay kit (E-BC-K773-M, Elabscience, Wuhan, China). The samples were homogenized and centrifuged, and the supernatant was collected. We conducted subsequent procedures according to the instructions provided in the kit manual.

### 2.8. Detection of Glutathione (GSH)

We employed a reduced GSH colorimetric assay kit (E-BC-K030-M, Elabscience) to quantify GSH levels in testes and Sertoli cells. Tissue and cell samples were homogenized in normal saline (0.9% NaCl) using either a Dounce homogenizer or an ultrasonic cell disruptor, and after centrifugation, the supernatant was collected for further analysis. DTNB solution and phosphate buffer were then added, and the mixture was vigorously stirred for 1 min and then left to stand for 5 min at room temperature. Subsequently, the optical density at 405 nm was measured using a spectrophotometer.

### 2.9. Determination of Lipid Peroxidation

Lipid peroxidation levels in the testes and cells were assessed using a lipid peroxidation MDA assay kit (S0131M, Beyotime, Shanghai, China), and the procedure was evaluated with a lipid peroxidation MDA assay kit (S0131M, Beyotime). The procedure was carried out according to the manufacturer’s instructions, following the collection of the supernatant from the tissue and cell samples. Absorbance was subsequently measured at 532 nm using a spectrophotometer.

### 2.10. Western-Blot Analysis

Whole-protein extracts from testis and Sertoli cells were lysed in RIPA lysis buffer supplemented with a 10% protease inhibitor cocktail, and we determined protein concentrations with a BCA Protein Assay Kit (P0012, Beyotime). For western-blot analysis, proteins from the lysates were separated by SDS-PAGE and transferred to PVDF membranes, and the membranes were incubated overnight at 4 °C with primary antibodies against ZO-1 (21773-1-AP, Proteintech, Wuhan, China), occludin (27260-1-AP, Proteintech, Wuhan, China), N-cadherin (22018-1-AP, Proteintech, Wuhan, China), β-catenin (51067-2-AP, Proteintech, Wuhan, China), Cx43 (A11752, ABclonal, Wuhan, China), EPS8 (43114S, CST, Boston, MA, USA), ARP3 (A4514, ABclonal, Wuhan, China), NCOA4 (PA5-96398, Invitrogen, Carlsbad, CA, USA), ACSL4 (22401-1-AP, Proteintech, Wuhan, China), FTH1 (381204, Zenbio, Chengdu, China), GPX4 (R381958, Zenbio, Chengdu, China), SLC7A11 (R382036, Zenbio, Chengdu, China), HO-1 (R24541, Zenbio, Chengdu, China), and β-actin (TA-09, ZSGB-Bio, Beijing, China)—using antibody dilutions at 1:1000. Afterward, the membranes were incubated with peroxidase-conjugated AffiniPure goat anti-rabbit or anti-mice secondary antibody at room temperature for 1 h. The expression levels of target protein bands were quantified using Image Lab software 6.1.0.

### 2.11. Statistical Analysis

An unpaired Student’s *t*-test or Welch’s *t*-test was employed based on the results of the homogeneity of variance test (F-test) to compare groups. For differences involving three or more groups, one-way analysis of variance (ANOVA) was utilized, followed by Tukey’s test for pairwise comparisons and Dunnett’s test for comparisons with a control group. All statistical analyses were carried out using SPSS 20.0, the data are presented as the mean ± standard deviation, with a significance level set at *p* < 0.05.

## 3. Results

### 3.1. Prepubertal Exposure to TCEP Leads to Testicular Developmental Toxicity

**Figure 1:**
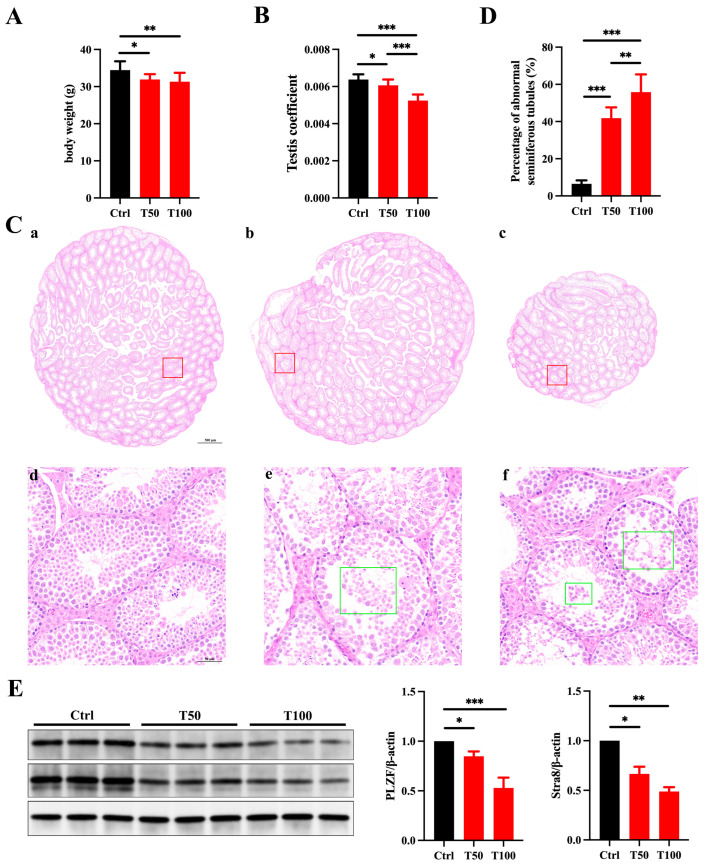
TCEP exposure leads to testicular toxicity. (**A**) The body weight in the three groups. (**B**) The testis coefficient in the three groups. (**C**) Testicular histomorphological changes in the three groups after H&E staining. (**a**,**d**) The control group. (**b**,**e**) The T50 group. (**c**,**f**) The T100 group. The red box delineates the magnified region, whereas the green box denotes the isolated germ cells. The scale bars in the top-row images represent 500 μm, and the scale bars in the bottom images represent 50 μm. (**D**) Percentages of abnormal seminiferous tubules with less than three layers of spermatogenic cells and/or sloughed germ cells in the three groups. (**E**) Expression levels of PLZF and Stra8 in the testes. * *p* < 0.05, ** *p* < 0.01, *** *p* < 0.001. Each experiment was replicated at least three times with independent samples.

Prepuberty is a pivotal phase in male reproductive system development, which is marked by unique changes in the testes that are crucial for establishing normal reproductive functions [23]. However, the toxic effects of TCEP exposure on testicular development during this crucial stage remain unexplored. In our study, prepubertal exposure to TCEP markedly reduced the body weight on PND35 and the organ coefficient of the testis, particularly in the T100 group (Figure 1A,B). H&E staining also revealed that TCEP exposure disrupted the structure of the testicular seminiferous tubules, with detached germ cells visible in the lumen and with a significant reduction in germ cell numbers (as shown in the green box); this phenotype was more deleterious in the T100 group relative to the T50 group (Figure 1C), and the percentage of seminiferous tubules displaying fewer than three layers of germ cells or evident structural abnormalities was significantly increased (Figure 1D). Furthermore, the expression levels of PLZF (a spermatogonia-specific marker) and Stra8 (a meiosis marker) were obviously reduced in TCEP-exposed testis (Figure 1E). These results demonstrated that TCEP exposure at the prepubertal stage induced testicular development toxicity.

### 3.2. TCEP Exposure Disrupts BTB Integrity

**Figure 2:**
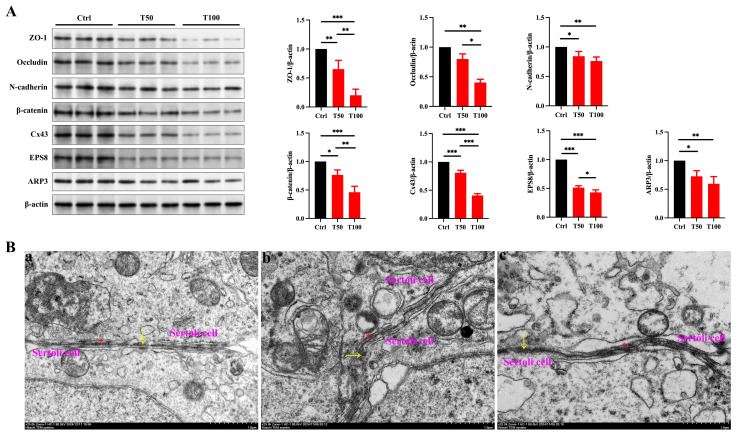
TCEP exposure disrupts BTB integrity. (**A**) Expression levels of BTB-associated proteins in the testes. (**B**) Assessment of BTB integrity using TEM. (**a**) The control group. (**b**) The T50 group. (**c**) The T100 group. The yellow arrows denoted the TJ; the red asterisks denoted the basal ES. * *p* < 0.05, ** *p* < 0.01, *** *p* < 0.001. Each experiment was replicated at least three times with independent samples.

Due to the critical role of Sertoli cells in testicular development—including forming the BTB and producing growth factors such as GDNF and FGF2 that promote germ cell development [24]—we hypothesized that TCEP caused testicular developmental toxicity by damaging Sertoli cells. Moreover, as the junctional structures between Sertoli cells are the primary targets of environmental pollutants, we were prompted to evaluate the integrity of the BTB. We first examined the expression of TJ proteins (ZO-1 and occludin), basal ES proteins (N-cadherin, β-catenin), and GJ proteins (Cx43) in the testis. Compared with the control group, the expression of all junctional proteins was notably reduced in the TCEP-exposed groups (Figure 2A). Additionally, as all of these junctional structures utilize actin microfilaments for their attachments, the bundling and branching changes in actin microfilaments thus determine the integrity of the junctional structures and BTB [25]. Actin-binding proteins (ABPs) EPS8 and ARP3 regulate actin microfilaments by promoting bundling and branching, respectively [26,27], and we found that the expression of these two ABPs was also attenuated after TCEP exposure (Figure 2A). TEM also showed that the TJs of the BTB were no longer compact but rather became looser (Figure 2B). The characteristic continuous “kissing points” of TJs (marked by yellow arrows) were replaced by discontinuous and disorganized strands, and the basal ES, typified by the actin filament bundles (marked by red asterisks), was also disrupted in the exposure groups. These results indicated that TCEP exposure impaired BTB integrity.

### 3.3. TCEP Exposure Induces Sertoli Cell Ferroptosis

**Figure 3:**
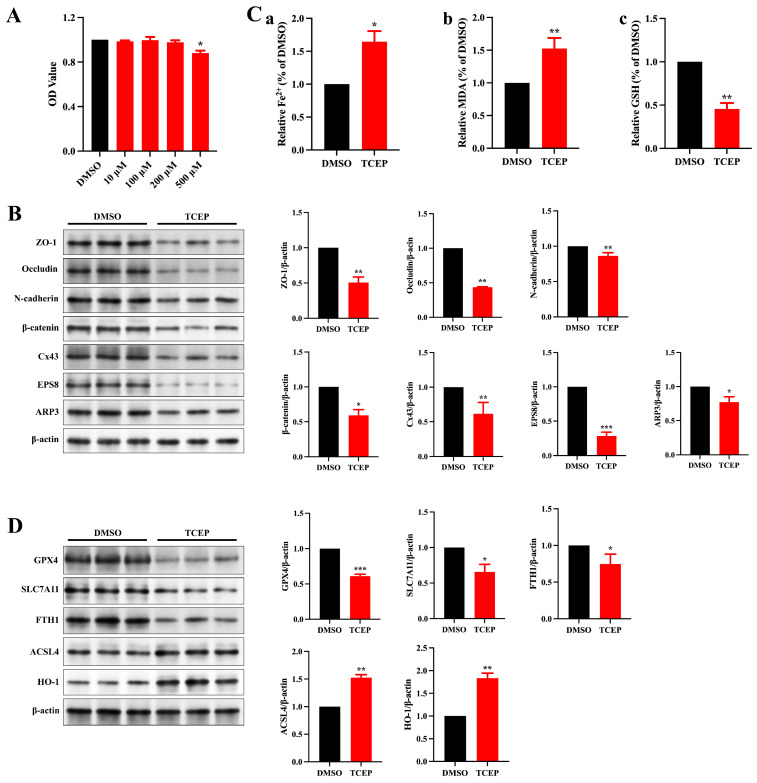
TCEP exposure leads to impairment of junctional function and ferroptosis in Sertoli cells. (**A**) Viability of Sertoli cells after TCEP exposure. (**B**) Expression levels of BTB-associated proteins in Sertoli cells. (**C**) Levels of ferrous iron, MDA, and GSH in Sertoli cells. (**a**) Level of ferrous iron. (**b**) Level of MDA. (**c**) Level of GSH. (**D**) Expression of ferroptosis-associated markers in Sertoli cells. * *p* < 0.05, ** *p* < 0.01, *** *p* < 0.001. Each experiment was replicated at least three times with independent samples.

We subsequently investigated the toxic effects of TCEP on Sertoli cells in order to identify the specific factor responsible for the disruption of the BTB. The viability of Sertoli cells was significantly diminished at the elevated TCEP concentration (Figure 3A), and a concentration of 500 μM was selected to perform the follow-up study. Congruent with the in vivo results, the expression of junctional proteins and ABPs fell significantly with TCEP (Figure 3B). PBDE-47 is also a flame retardant like TCEP, and the former has been shown to disrupt BTB integrity by inducing ferroptosis in Sertoli cells; we therefore hypothesized that TCEP likewise stimulated ferroptosis in Sertoli cells. The levels of Fe^2+^ and MDA were much higher and that of GSH was much lower in the TCEP group compared to the DMSO group (Figure 3C). Additionally, after TCEP exposure, the expression of HO-1 (an anti-oxidative protein) was elevated; that of ferroptosis marker ACSL4 was increased; and that of SLC7A11, GPX4, and FTH1 was decreased (Figure 3D). These results suggested that TCEP exposure resulted in Sertoli cell ferroptosis.

### 3.4. TCEP Exposure Impairs Sertoli Cell Junctional Functions via Ferroptosis

**Figure 4:**
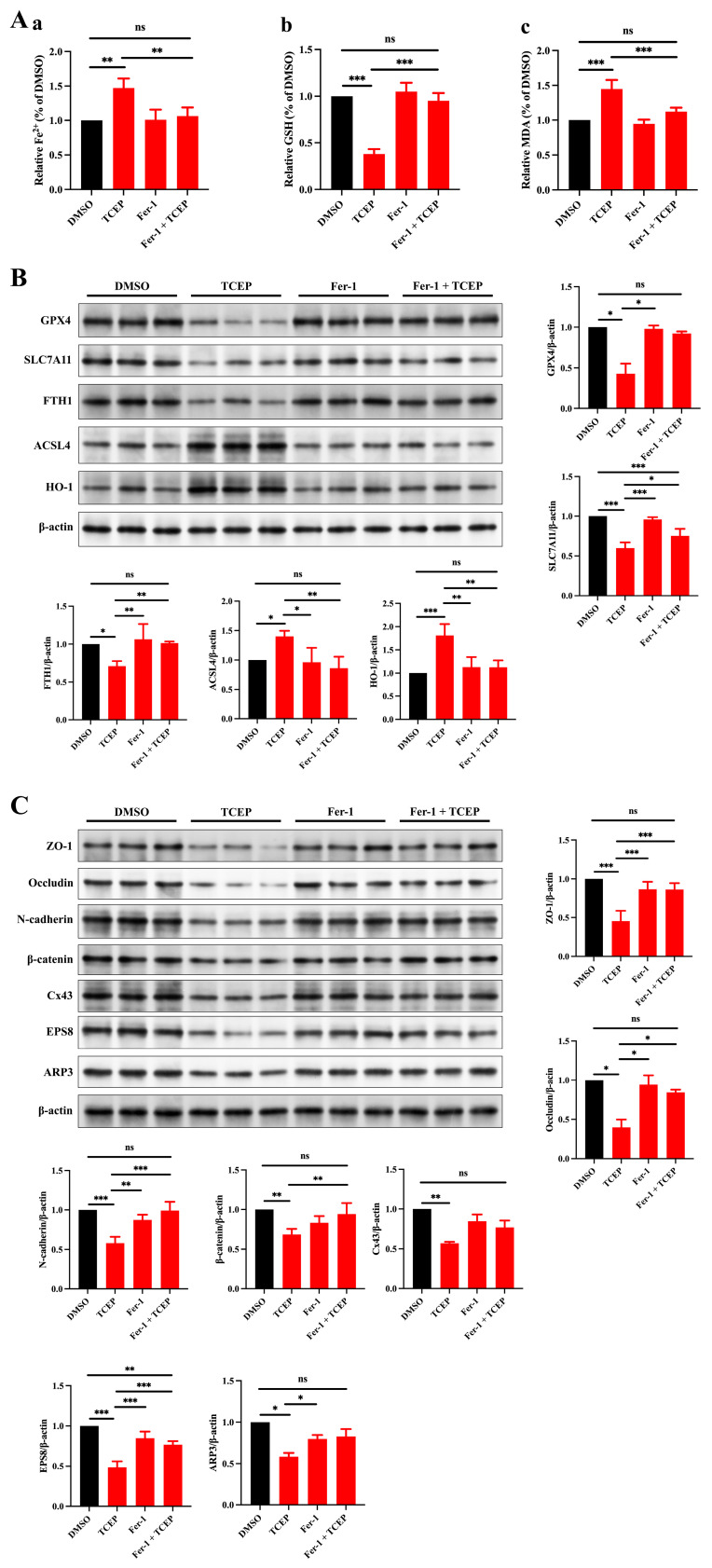
Treatment with Fer-1 ameliorates the TCEP-induced impairment of junctional functionality in Sertoli cells. (**A**) Levels of ferrous iron, MDA, and GSH in Sertoli cells after Fer-1 treatment. (**a**) Level of ferrous iron. (**b)** Level of MDA. (**c**) Level of GSH. (**B**) Expression of ferroptosis-associated markers in Sertoli cells after Fer-1 treatment. (**C**) Expression levels of BTB-associated proteins in Sertoli cells after Fer-1 treatment. * *p* < 0.05, ** *p* < 0.01, *** *p* < 0.001. Each experiment was replicated at least three times with independent samples.

To confirm a direct link between TCEP-induced ferroptosis in Sertoli cells and impairment of BTB integrity, we deployed ferrostatin-1 (Fer-1) to inhibit ferroptosis. We discerned that Fer-1 treatment reversed the alterations in the levels of Fe^2+^, MDA, and GSH (Figure 4A), the expression of HO-1 and ferroptosis markers (Figure 4B), and the expression of junctional proteins and ABPs (Figure 4C), thus demonstrating that Fer-1 can rescue Sertoli cell ferroptosis and reconstruct BTB integrity.

### 3.5. TCEP Induces Ferroptosis in Sertoli Cells and the Testis via Ferritinophagy

**Figure 5:**
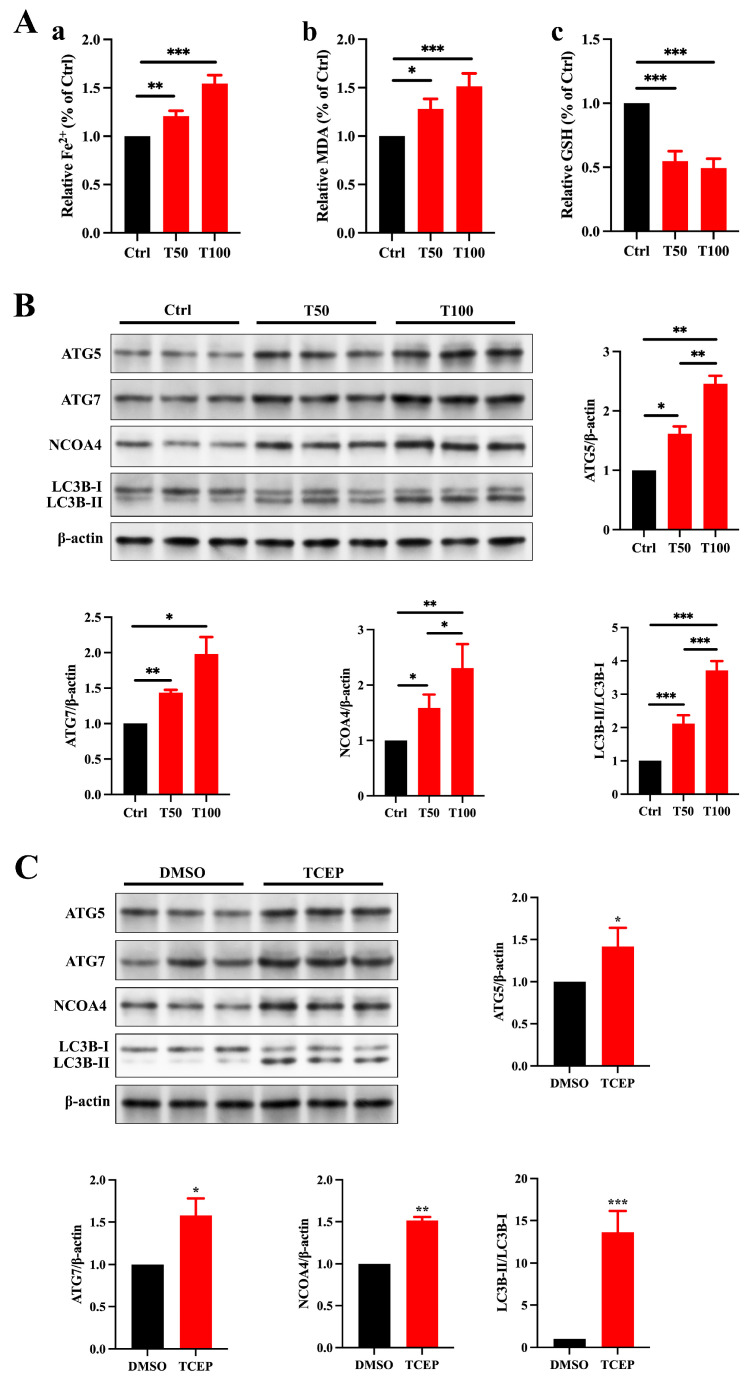
TCEP induces ferroptosis in Sertoli cells and testis through ferritinophagy. (**A**) Levels of ferrous iron, MDA, and GSH in the testes. (**a**) Level of ferrous iron. (**b**) Level of MDA. (**c**) Level of GSH. (**B**) Ferritinophagic activity in the testes. (**C**) Ferritinophagic activity in Sertoli cells. * *p* < 0.05, ** *p* < 0.01, *** *p* < 0.001. Each experiment was replicated at least three times with independent samples.

We then determined the level of ferroptosis in the TCEP-exposed testis and showed that TCEP exposure similarly enhanced the levels of Fe^2+^ and MDA and the expression of ACSL4 and inhibited the levels of GSH in the testis (Figure 5A). In addition, after TCEP exposure, the expression of HO-1 was enhanced; that of the ferroptosis marker ACSL4 was increased; and that of SLC7A11, GPX4, and FTH1 was decreased (Figure 5B). These results indicated that TCEP exposure led to ferroptosis in the testis.

Ferritinophagy is a selective form of autophagy that plays a crucial role in regulating intracellular iron metabolism, facilitated by NCOA4. This process involves the transport of ferritin into autophagolysosomes for degradation, resulting in the release of free iron. When ferritinophagy is overly activated, NCOA4 promotes the autophagic breakdown of ferritin, releasing an excess of free iron, which can trigger ferroptosis in cells [27]. Based on the reduced expression of FTH1 in the testes and Sertoli cells exposed to TCEP, we hypothesize that ferritinophagy is involved in regulating the process by which TCEP disrupts BTB integrity. The expression of ATG5, ATG7, NCOA4, and LC3B II/I was much higher in TCEP-exposed testicular and Sertoli cells than that in the control group (Figure 5C), signifying that TCEP disrupted BTB integrity through ferritinophagy-mediated ferroptosis.

## 4. Discussion

While OPFRs have been extensively utilized as alternatives to PBDEs in applications such as flame retardants, plasticizers, and anti-foaming agents [28], their applications are often associated with toxicity—highlighting the importance of assessing their potential health and environmental impacts [29]. The amount of OPFRs consumed worldwide was 186,000 tons in 2001, which increased to over 1 million tons by 2018 [30]. As OPFRs are incorporated into products as physical additives, they are easily released into the surrounding environment through volatilization during the production, use, and recycling stages. OPFRs are presently detected in virtually all environmental media, such as air, water, soil, sediment, and living organisms [31]. As a result, their harmful effects have garnered increasing attention. Human exposure to OPFRs mainly occurs through three pathways: inhaling dust or vapors, ingesting contaminated food or water, and dermal absorption from direct contact with products containing these chemicals. Additionally, environmental release into air, water, or soil—followed by accumulation in food chains—further contributes to human exposure [32]. TCEP is a typical chlorinated OPFR and is more persistent than other nonhalogenated alkyl- or aryl-OPFRs [33]. Thus, a deeper understanding of the molecular toxic effects of TCEP is crucial in identifying biomarkers with respect to exposure and effects, improving risk-assessment models, and developing targeted therapeutic and preventive strategies.

A decline in human fertility rates in recent years has fostered increased attention to the impact of environmental pollutants on reproduction. Several animal studies have revealed that exposure to TCEP precipitated spermatogenic dysfunction [34], abnormal testicular structures, oxidative and endocrine disruption [15], and steroidogenic alterations in Leydig cells [16]. However, the mechanisms underlying TCEP-induced testicular toxicity remain arcane. Notably, Sertoli cells (which play a crucial role in supporting testicular development and maintaining normal spermatogenesis [35]), have not been reported to be affected by TCEP.

Sertoli cells portray a vital role in supporting spermatogenesis and preserving the immune balance within the testicular microenvironment. By forming the BTB, these cells establish an immune-privileged zone essential for germ cell development [36]. Moreover, these cells release a range of cytokines and growth factors that not only promote germ cell survival and differentiation but also modulate local immune activity so as to safeguard germ cells from immune-related damage [37]. Various environmental toxicants, including di(2-ethylhexyl) phthalate (DEHP), particulate matter ≤ 2.5 µm in size (PM2.5), bisphenol S (BPS), microplastics (MPs), and PBDE-47, have been demonstrated to impair testicular development and spermatogenesis by damaging Sertoli cells and compromising the integrity of the BTB [20,38,39,40]. We, therefore, proposed that the impairment of Sertoli cells and the disruption of BTB integrity were important to the testicular toxicity induced by TCEP.

The integrity of the BTB was the primary focus of our investigation, as the junctional structures between Sertoli cells comprise the chief targets of environmental toxicant action. Since the BTB is formed by PND21 [35], the prepubertal stage is critical to the development of the male reproductive system and is marked by an increased sensitivity to sex steroids [41]. Any disruption in testicular development during this period could result in negative effects that may become apparent during puberty or may even persist into adulthood. [42,43]. We thus exposed mice to TCEP from PND22 to PND35, and the exposure dose was selected based on previously published reports [3,15]. As expected, TCEP exposure disrupted junctional functionality in the testis and Sertoli cells. We observed that the expression of all BTB-associated proteins was significantly reduced, and TEM revealed the destruction of the TJs. This is the first-ever study to show that TCEP induces BTB disruption. Additionally, the decreased expression of ABP suggested that actin microfilaments were disorganized after TCEP exposure. Actin microfilaments play a significant role not only in regulating BTB integrity but also in phagocytosing dead germ cells by forming phagocytic cups; in fact, over 75% of germ cells undergo apoptosis, with Sertoli cells rapidly phagocytosing the apoptotic cells during spermatogenesis [44]. If these apoptotic germ cells are not cleared promptly, immunogenic components are released, potentially triggering testicular inflammation that can result in testicular damage and impair spermatogenesis [45]. We noted that there were dead germ cells in the seminiferous tubules, suggesting that phagocytosis of Sertoli cells was impaired by TCEP; however, this requires further investigation.

The majority of environmental toxicants induce BTB disruption, testicular developmental toxicity, and abnormal spermatogenesis through oxidative stress, and TCEP has also been shown to induce oxidative stress and cause damage to the liver [46], lungs [47], and nerves [48]; and it can similarly induce oxidative stress damage in the testis [15]. We also found that the expression of HO-1 was much higher after TCEP exposure. Since oxidative stress is closely related to ferroptosis, we evaluated this programmed cell-death pathway in the testis and Sertoli cells and demonstrated that TCEP disrupted BTB integrity Via stimulation of ferroptosis by our application of Fer-1. It was noted that TCEP exposure led to a significant increase in MDA and a decline in glutathione; viability of Sertoli cells, however, was relatively slightly affected; this may be due to the fact that the exposure duration may not have exceeded the threshold required to trigger widespread cell death. Moreover, our results aligned with previous studies, demonstrating that during MEHP-induced ferroptosis in TM4 cells, the extent of cell death remains relatively low, while MDA levels exhibit an obvious increase [49]. Additionally, TCEP exposure may activate antioxidant-related genes in Sertoli cells (e.g., Nrf2, Cat, and Nqo1) to mitigate oxidative damage and cell death. This has been confirmed in other studies [50].

FTH1 is a crucial regulator of iron storage and release, playing a key role in maintaining iron homeostasis by sequestering excess free iron within ferritin [22]. This function enables FTH1 to mitigate ferroptosis by limiting free iron availability, reducing ROS generation, preventing lipid peroxidation, and sustaining iron balance [51]. Growing evidence suggests that the activation of ferritinophagy leads to the degradation of FTH1 in various pathological conditions, including neuroinflammation [52], lung injury [53], and atherosclerosis [54]. Additionally, exposure to TCEP resulted in a significant reduction in FTH1 expression in both in vivo and in vitro studies. Based on these findings, we hypothesize that ferritinophagy-mediated FTH1 degradation is a critical element in the ferroptosis of Sertoli cells and the disruption of BTB integrity induced by TCEP. Our study thus revealed the activation of ferritinophagy in testes and Sertoli cells exposed to TCEP. Although we did not use an autophagy inhibitor to further validate the critical role of ferritinophagy, the restoration of BTB integrity through Fer-1 intervention, along with the expression levels of NCOA4 and FTH1 observed both in vivo and in vitro, indicated that the activity of ferritinophagy was enhanced following TCEP exposure. Furthermore, we will utilize siRNA to knock down the expression of Ncoa4 in our future studies.

In this study, we observed that TCEP exposure led to a marked increase in NCOA4 expression that subsequently enhanced the degradation of FTH1 Via ferritinophagy. However, additional research is warranted so as to ascertain whether inhibiting NCOA4-mediated ferritinophagy Via Ncoa4 knockdown or knockout can prevent iron accumulation and ferroptosis induced by TCEP. We suggest that future studies also explore the effects of combined exposure to TCEP with other types of OPFRs or environmental toxicants on Sertoli cells.

## 5. Conclusions

In summary, our findings revealed that ferritinophagy-mediated degradation of FTH1 triggered ferroptosis in Sertoli cells, which in turn disrupted BTB integrity and generated testicular developmental toxicity in response to TCEP exposure. These results offer novel insights into ferroptosis as a mechanism underlying TCEP-induced testicular abnormalities and suggest potential targets for mitigating TCEP-related subfertility and infertility in males.

## Data Availability

The data that support the findings of this study are available from the corresponding author upon reasonable request.

## References

[1] Blum A., Behl M., Birnbaum L.S., Diamond M.L., Phillips A., Singla V., Sipes N.S., Stapleton H.M., Venier M. (2019). Organophosphate Ester Flame Retardants: Are They a Regrettable Substitution for Polybrominated Diphenyl Ethers?. Environ. Sci. Technol. Lett..

[2] Lee S., Jeong W., Kannan K., Moon H.-B. (2016). Occurrence and exposure assessment of organophosphate flame retardants (OPFRs) through the consumption of drinking water in Korea. Water Res..

[3] Fang B., Wang C., Du X., Sun G., Jia B., Liu X., Qu Y., Zhang Q., Yang Y., Li Y.Q. (2024). Structure-dependent destructive adsorption of organophosphate flame retardants on lipid membranes. J. Hazard. Mater..

[4] Yang J., Li X., Yang H., Zhao W., Li Y. (2022). OPFRs in e-waste sites: Integrating in silico approaches, selective bioremediation, and health risk management of residents surrounding. J. Hazard. Mater..

[5] Ma H., He J., Fan H., Zhang N., Wu Q., Zhang S., Zhang C., Huang T., Gao H., Ma J. (2024). The influence of emerging atmospheric organophosphorus flame retardants from land source emissions on the East China Sea. J. Hazard. Mater..

[6] Castro-Jiménez J., González-Gaya B., Pizarro M., Casal P., Pizarro-Álvarez C., Dachs J. (2016). Organophosphate Ester Flame Retardants and Plasticizers in the Global Oceanic Atmosphere. Environ. Sci. Technol..

[7] Mendy A., Percy Z., Braun J.M., Lanphear B., La Guardia M.J., Hale R.C., Yolton K., Chen A. (2024). Prenatal and postnatal exposure to organophosphate esters and replacement flame retardant mixtures and childhood respiratory outcomes. Environ. Res..

[8] Win-Shwe T.T., Yanagisawa R., Lwin T.T., Kawakami F., Koike E., Takano H. (2022). Dietary Exposure to Flame Retardant Tris (2-Butoxyethyl) Phosphate Altered Neurobehavior and Neuroinflammatory Responses in a Mouse Model of Allergic Asthma. Int. J. Mol. Sci..

[9] Zhang B., Lu S., Huang M., Zhou M., Zhou Z., Zheng H., Jiang Y., Bai X., Zhang T. (2018). Urinary metabolites of organophosphate flame retardants in 0-5-year-old children: Potential exposure risk for inpatients and home-stay infants. Environ. Pollut..

[10] Li K., Qi Z., Xie Z., Li W., Yang X., Zhai Y., Zhou X., Xie X., Song W. (2024). TDMPP activation of estrogen receptor 2a regulates smc2 and p53 signaling to interfere with liver development in zebrafish (Danio rerio). J. Hazard. Mater..

[11] Tsai K.-F., Cheng F.-J., Huang W.-T., Kung C.-T., Lee C.-T., Cheng B.-C., Chen J.-B., Li S.-H., Wang C.-C., Wang L.-J. (2022). The associations between renal disease severity and exposure to organophosphate flame retardants in patients with chronic kidney disease. Environ. Int..

[12] Cui H., Chang Y., Jiang X., Li M. (2020). Triphenyl phosphate exposure induces kidney structural damage and gut microbiota disorders in mice under different diets. Environ. Int..

[13] Chiang C., Mahalingam S., Flaws J.A. (2017). Environmental Contaminants Affecting Fertility and Somatic Health. Semin. Reprod. Med..

[14] Wang H., Ding J., Luo S., Yan M., Hu F. (2024). Unveiling the mechanisms of reproductive toxicity induced by full life-cycle exposure to environmentally relevant concentrations of tris(2-chloroethyl) phosphate in male zebrafish. Aquat. Toxicol..

[15] Chen G., Jin Y., Wu Y., Liu L., Fu Z. (2015). Exposure of male mice to two kinds of organophosphate flame retardants (OPFRs) induced oxidative stress and endocrine disruption. Environ. Toxicol. Pharmacol..

[16] Chen G., Zhang S., Jin Y., Wu Y., Liu L., Qian H., Fu Z. (2015). TPP and TCEP induce oxidative stress and alter steroidogenesis in TM3 Leydig cells. Reprod. Toxicol..

[17] Washburn R.L., Hibler T., Kaur G., Dufour J.M. (2022). Sertoli Cell Immune Regulation: A Double-Edged Sword. Front. Immunol..

[18] Li N., Mruk D.D., Lee W.M., Wong C.K., Cheng C.Y. (2016). Is toxicant-induced Sertoli cell injury in vitro a useful model to study molecular mechanisms in spermatogenesis?. Semin. Cell Dev. Biol..

[19] Gao Y., Mruk D.D., Cheng C.Y. (2015). Sertoli cells are the target of environmental toxicants in the testis—A mechanistic and therapeutic insight. Expert Opin. Ther. Targets.

[20] Huang X., Fu Y., Wang S., Guo Q., Wu Y., Zheng X., Wang J., Wu S., Shen L., Wei G. (2024). 2,2′,4,4′-Tetrabromodiphenyl ether exposure disrupts blood-testis barrier integrity through CMA-mediated ferroptosis. Sci. Total Environ..

[21] Jiang X., Stockwell B.R., Conrad M. (2021). Ferroptosis: Mechanisms, biology and role in disease. Nat. Rev. Mol. Cell Biol..

[22] Santana-Codina N., Gikandi A., Mancias J.D. (2021). The Role of NCOA4-Mediated Ferritinophagy in Ferroptosis. Adv. Exp. Med. Biol..

[23] Allen C.M., Lopes F., Mitchell R.T., Spears N. (2018). How does chemotherapy treatment damage the prepubertal testis?. Reproduction.

[24] Kanatsu-Shinohara M., Shinohara T. (2013). Spermatogonial stem cell self-renewal and development. Annu. Rev. Cell Dev. Biol..

[25] Li N., Tang E.I., Cheng C.Y. (2016). Regulation of blood-testis barrier by actin binding proteins and protein kinases. Reproduction.

[26] Lie P.P., Mruk D.D., Lee W.M., Cheng C.Y. (2009). Epidermal growth factor receptor pathway substrate 8 (Eps8) is a novel regulator of cell adhesion and the blood-testis barrier integrity in the seminiferous epithelium. FASEB J..

[27] Lie P.P., Chan A.Y., Mruk D.D., Lee W.M., Cheng C.Y. (2010). Restricted Arp3 expression in the testis prevents blood-testis barrier disruption during junction restructuring at spermatogenesis. Proc. Natl. Acad. Sci. USA.

[28] Qadeer A., Mubeen S., Liu M., Bekele T.G., Ohoro C.R., Adeniji A.O., Alraih A.M., Ajmal Z., Alshammari A.S., Al-Hadeethi Y. (2024). Global environmental and toxicological impacts of polybrominated diphenyl ethers versus organophosphate esters: A comparative analysis and regrettable substitution dilemma. J. Hazard. Mater..

[29] Hoehn R.M., Jahl L.G., Herkert N.J., Hoffman K., Soehl A., Diamond M.L., Blum A., Stapleton H.M. (2024). Flame Retardant Exposure in Vehicles Is Influenced by Use in Seat Foam and Temperature. Environ. Sci. Technol..

[30] Fu J., Fu K., Hu B., Zhou W., Fu Y., Gu L., Zhang Q., Zhang A., Fu J., Jiang G. (2023). Source Identification of Organophosphate Esters through the Profiles in Proglacial and Ocean Sediments from Ny-Ålesund, the Arctic. Environ. Sci. Technol..

[31] Yan Z., Feng C., Leung K.M., Luo Y., Wang J., Jin X., Wu F. (2023). Insights into the geographical distribution, bioaccumulation characteristics, and ecological risks of organophosphate esters. J. Hazard. Mater..

[32] Yang J., Zhao Y., Li M., Du M., Li X., Li Y. (2019). A Review of a Class of Emerging Contaminants: The Classification, Distribution, Intensity of Consumption, Synthesis Routes, Environmental Effects and Expectation of Pollution Abatement to Organophosphate Flame Retardants (OPFRs). Int. J. Mol. Sci..

[33] Yang T., Zhou X., Wu Y., Liang Y., Zeng X., Yu Z. (2024). Metagenomic analyses of aerobic bacterial enrichment cultures that degraded Tris(2-chloroethyl) phosphate (TCEP) and its transformation products. Environ. Pollut..

[34] (1997). Reproductive toxicology. Tris(2-chloroethyl)phosphate. Environ. Health Perspect..

[35] Cheng C.Y., Mruk D.D. (2012). The blood-testis barrier and its implications for male contraception. Pharmacol. Rev..

[36] Li N., Mruk D.D., Cheng C.Y. (2015). Actin binding proteins in blood-testis barrier function. Curr. Opin. Endocrinol. Diabetes Obes..

[37] Chen R., Wang F., Chen Y., Han D. (2022). Immune homeostasis and disorder in the testis—Roles of Sertoli cells. J. Reprod. Immunol..

[38] Wang J., Wei Y., Wu Y., Zhao T., Kang L., Han L., Chen J., Long C., Wei G., Shen L. (2024). Di-(2-ethylhexyl) phthalate induces prepubertal testicular injury through MAM-related mitochondrial calcium overload in Leydig and Sertoli cell apoptosis. Toxicology.

[39] Wu H., Wei Y., Zhou Y., Long C., Hong Y., Fu Y., Zhao T., Wang J., Wu Y., Wu S. (2021). Bisphenol S perturbs Sertoli cell junctions in male rats via alterations in cytoskeletal organization mediated by an imbalance between mTORC1 and mTORC2. Sci. Total Environ..

[40] Wei Y., Zhou Y., Long C., Wu H., Hong Y., Fu Y., Wang J., Wu Y., Shen L., Wei G. (2021). Polystyrene microplastics disrupt the blood-testis barrier integrity through ROS-Mediated imbalance of mTORC1 and mTORC2. Environ. Pollut..

[41] Perobelli J.E. (2014). The male peripubertal phase as a developmental window for reproductive toxicology studies. Curr. Pharm. Des..

[42] van den Driesche S., Kilcoyne K.R., Wagner I., Rebourcet D., Boyle A., Mitchell R., McKinnell C., Macpherson S., Donat R., Shukla C.J. (2017). Experimentally induced testicular dysgenesis syndrome originates in the masculinization programming window. JCI Insight.

[43] Kilcoyne K.R., Mitchell R.T. (2019). Effect of environmental and pharmaceutical exposures on fetal testis development and function: A systematic review of human experimental data. Hum. Reprod. Update.

[44] Shi J., Gao S., Chen Z., Chen Z., Yun D., Wu X., Sun F. (2023). Absence of MerTK disrupts spermatogenesis in an age-dependent manner. Mol. Cell. Endocrinol..

[45] Wang H., Wang H., Xiong W., Chen Y., Ma Q., Ma J., Ge Y., Han D. (2006). Evaluation on the phagocytosis of apoptotic spermatogenic cells by Sertoli cells in vitro through detecting lipid droplet formation by Oil Red O staining. Reproduction.

[46] Hu F., Li W., Wang H., Peng H., He J., Ding J., Zhang W. (2023). Environmentally relevant concentrations of tris (2-chloroethyl) phosphate (TCEP) induce hepatotoxicity in zebrafish (Danio rerio): A whole life-cycle assessment. Fish Physiol. Biochem..

[47] Meng Y., Xu X., Niu D., Xu Y., Qiu Y., Zhu Z., Zhang H., Yin D. (2022). Organophosphate flame retardants induce oxidative stress and Chop/Caspase 3-related apoptosis via Sod1/p53/Map3k6/Fkbp5 in NCI-1975 cells. Sci. Total Environ..

[48] Wang C., Chen Z., Lu Y., Wang L., Zhang Y., Zhu X., Song J. (2020). Neurotoxicity and related mechanisms of flame retardant TCEP exposure in mice. Toxicol. Mech. Methods.

[49] Wu Y., Wang J., Zhao T., Chen J., Kang L., Wei Y., Han L., Shen L., Long C., Wu S. (2022). Di-(2-ethylhexyl) phthalate exposure leads to ferroptosis via the HIF-1α/HO-1 signaling pathway in mouse testes. J. Hazard. Mater..

[50] Peng H., Wang H., Li W., Jing C., Zhang W., Zhao H., Hu F. (2023). Life-cycle exposure to tris (2-chloroethyl) phosphate (TCEP) causes alterations in antioxidative status, ion regulation and histology of zebrafish gills. Comp. Biochem. Physiol. Toxicol. Pharmacol. CBP.

[51] Muhoberac B.B., Vidal R. (2019). Iron, Ferritin, Hereditary Ferritinopathy, and Neurodegeneration. Front. Neurosci..

[52] Sui H., Sun Z., Liu C., Xi H. (2024). Ferritinophagy promotes microglia ferroptosis to aggravate neuroinflammation induced by cerebral ischemia-reperfusion injury via activation of the cGAS-STING signaling pathway. Neurochem. Int..

[53] Ou H., Lin J., Ji L., Ye L., Ling M., Liao X., Lin F., Wang Y., Luo B., Hu Z. (2024). Ferritinophagy mediated by the AMPK/ULK1 pathway is involved in ferroptosis subsequent to ventilator-induced lung injury. Respir. Res..

[54] Zhu L., Liu Z., Liu J., Li Z., Bao Y., Sun X., Zhao W., Zhou A., Wu H. (2024). NCOA4 linked to endothelial cell ferritinophagy and ferroptosis:a key regulator aggravate aortic endothelial inflammation and atherosclerosis. Redox Biol..

